# Gold Nanoparticles Radio-Sensitize and Reduce Cell Survival in Lewis Lung Carcinoma

**DOI:** 10.3390/nano10091717

**Published:** 2020-08-30

**Authors:** Arvind Pandey, Veronica Vighetto, Nicola Di Marzio, Francesca Ferraro, Matteo Hirsch, Nicola Ferrante, Sankar Mitra, Alessandro Grattoni, Carly S. Filgueira

**Affiliations:** 1 Department of Radiation Oncology, Houston Methodist Research Institute, Houston, TX 77030, USA; apandey@houstonmethodist.org (A.P.); smitra2@houstonmethodist.org (S.M.); agrattoni@houstonmethodist.org (A.G.); 2 Department of Nanomedicine, Houston Methodist Research Institute, Houston, TX 77030, USA; veronica@vighetto.it (V.V.); nicola.dimarzio@studenti.polito.it (N.D.M.); frafer92f@libero.it (F.F.); matteo.hirsch@gmail.com (M.H.); nicola.ferrante@studenti.polito.it (N.F.); 3 Department of Applied Science and Technology, Politecnico di Torino, 10129 Torino, Italy; 4 Department of Electronic and Telecommunications, Politecnico di Torino, 10129 Torino, Italy; 5 Department of Biomedical Engineering, Politecnico di Torino, 10129 Torino, Italy; 6 Department of Surgery, Houston Methodist Research Institute, Houston, TX 77030, USA; 7 Department of Cardiovascular Surgery, Houston Methodist Research Institute, Houston, TX 77030, USA

**Keywords:** gold nanoparticles, Lewis lung carcinoma, radio-sensitization, clonogenic assay, comet assay

## Abstract

It has been suggested that particle size plays an important role in determining the genotoxicity of gold nanoparticles (GNPs). The purpose of this study was to compare the potential radio-sensitization effects of two different sized GNPs (3.9 and 37.4 nm) fabricated and examined in vitro in Lewis lung carcinoma (LLC) as a model of non-small cell lung cancer through use of comet and clonogenic assays. After treatment with 2Gy X-ray irradiation, both particle sizes demonstrated increased DNA damage when compared to treatment with particles only and radiation alone. This radio-sensitization was further translated into a reduction in cell survival demonstrated by clonogenicity. This work indicates that GNPs of both sizes induce DNA damage in LLC cells at the tested concentrations, whereas the 37.4 nm particle size treatment group demonstrated greater significance in vitro. The presented data aids in the evaluation of the radiobiological response of Lewis lung carcinoma cells treated with gold nanoparticles.

## 1. Introduction

Gold nanoparticles (GNPs) offer a means to transport agents to diseased cells or tissues because of their physical, chemical, and optical properties which are specifically dependent on size, adaptability, and biocompatibility [1]. They can also act as cancer therapeutics and diagnostic tools, and have been demonstrated as novel molecular imaging contrast agents for computed tomography (CT) imaging, and photothermal cancer therapy [2,3]. The high atomic number of elemental gold (Z = 79) compared to that of soft tissues, permits metal enhanced radiotherapy, where their presence can amplify delivered ionizing radiation. Current theory suggests that gold nanoparticle-mediated radio-sensitization is a combination of physical, chemical, and biological effects. On the physical side, multiple effects occur, including the generation of photoelectrons, Auger electrons, and low-energy secondary electrons. These emissions produce ionization effects in the neighboring tissues [4]. In addition, GNP-mediated radio-enhancement is likely due to modulation in the cell cycle and increased production of reactive oxygen species (ROS), where enhanced localized absorption of X-rays results in energy deposition in the form of free radicals and electrons, causing cell damage [5]. However, it is still unclear how significantly a difference in particle size can affect the degree of radio-enhancement, since size can affect cellular uptake. Leung et al. [6] used Monte Carlo simulation and showed that GNPs of greater sizes increase the generation of secondary electrons. Experimentally, Brun et al. [7] concluded that with a constant effective X-ray energy of 49 keV, larger sized GNPs (92 nm) were more efficient radio-sensitizers than those of smaller diameter (8 nm). However, Butterworth et al. [8] found that, for smaller (5 and 20 nm) particles, the chemical yields of DNA damage in irradiated samples were significantly greater than that for 1.5 μm particles. Because of the reported discrepancy in size in the literature, we assessed two different-sized GNPs as radio-sensitizers in lung cancer cells.

Here, we exploit GNPs because of their desirable optical and electronic properties, which make them an excellent absorber of X-rays. We sought to assess the in-vitro radiobiological response of Lewis lung carcinoma (LLC) treated with low dose (2 Gy) X-ray irradiation alone and in combination with two different-sized gold nanoparticles to evaluate GNP-mediated effects. The aim of this study was to determine if GNPs result in greater DNA damage in lung cancer cells in the presence of irradiation and if modifying particle size resulted in differences in the radiobiological response.

## 2. Materials and Methods 

### 2.1. Synthesis of Gold Nanoparticles (GNPs)

Small GNPs (SGNP) were synthesized according to Duff et al. [9] using tetrakis(hydroxymethyl)phosphonium chloride (Sigma–Aldrich, St. Louis, MO, USA, 404861) and hydrogen tetrachloroaurate(III) hydrate (Sigma–Aldrich, St. Louis, MO, USA, 254169). Particles were also observed and measured on a Bruker Multimode Atomic Force Microscope (AFM) to yield a size of approximately 3.86 ± 1.27 nm. Z-potential values of −55.6 ± 13.5 mV were obtained, which confirm the presence of a layer of absorbed citrate anions. Citrate stabilizes the particles, minimizing aggregation. These anions can be displaced by wet chemistry to fabricate highly ordered arrays [10], self-assembled monolayers, [11] or hybrid lipid bilayers [12].

Big GNPs (BGNP) were synthesized using citric acid (Sigma, St. Louis, MO, USA, C3674) and gold (III) chloride (Sigma, 379948). Briefly, 600 μL of MilliQ was added to an Erlenmeyer flask and placed on a hot plate until vigorous boiling. After 30 s of refluxing, 4.8 mL of 0.039 M aqueous citrate was added to the flask. Finally, after about one minute, 7 mL of 0.033 M gold (III) chloride was rapidly added to the boiling solution and left on the hot plate for four minutes until the observed color change was complete. The solution equilibrated to room temperature and was stored for further use. Dynamic light scattering (DLS) was used to rapidly and qualitatively size the particles and obtain a polydispersity index (PDI) and Zeta Potential was measured (Malvin). The solutions yield Z-potentials of −40.0 ± 6.0 mV (5 replicates in 10 mM KCl solution where the result is reported as mean ± SD). Particles were also observed and measured on a FEI Nova NanoSEM 230 (FEI Co., Hillsboro, Oregon, USA) and a JEOL 1230 High Contrast TEM (JEOL, Peabody, MA, USA), yielding an average particle diameter of 37.39 ± 5.52 nm.

### 2.2. Cell Culture

Murine Lewis lung carcinoma (LLC) cells were obtained from ATCC^®^ (American Type Culture Collection, Manassas, VA, USA) and subcultured according to manufacturer’s recommended protocols, where the complete growth medium consisted of DMEM with 10% FBS and subcultures were prepared by diluting the suspensions 1:4 to 1:6 using 0.25% trypsin–0.53 mM EDTA solution (Thermo Fisher Scientific, Waltham, MA, USA). Cells were made to express luciferase by the use of plasmid pLenti PGK V5-LUC Neo [13] (Addgene, Cambridge, MA, USA) which was packaged in lentiviral particles. The packaging was performed at the Baylor College of Medicine (BCM) vector core facility. The plasmid was transfected into Human Embryonic Kidney (HEK-293T) cells, and the conditioned media collected and used to infect the LLC1 for 24 h. After 24 h, selection was initiated with G418 (Geneticin, ThermoFisher Scientific, Waltham, MA, USA). Dulbecco’s Modified Eagle’s Medium (DMEM, ATCC^®^, Manassas, VA, USA) was made complete by adding 10% fetal bovine serum (FBS, USDA approved, ATCC^®^, Manassas, VA, USA) and 1% Geneticin™ (ThermoFisher Scientific, Waltham, MA, USA) for the luciferase-expressing cells to maintain culture. Cells were kept at 37 °C and 5% humidity in HERAcell 150i CO_2_ incubator (ThermoFisher Scientific, Waltham, MA, USA).

### 2.3. Comet Assay

A neutral comet assay was performed using a CometAssay^®^ Kit (Trevigen, Gaithersburg, MD, USA), as per manufacturer’s instructions, to detect DNA damage due to irradiation. Briefly LLC were incubated with 54.5 µg of either SGNP or BGNP for 48 h and irradiated with 2 Gy using a Rad Source RS-2000 Biological Research Irradiator (Rad Source Technologies, Buford, GA, USA). Thirty minutes after irradiation cells were collected, counted, and mixed with 0.5% low melting point (LMP) agarose and spread over the comet slide. Slides were then immersed in an ice-cold freshly prepared lysis solution for at least 1 h. The slides were taken out of the lysis solution and then placed in a cold 1x neutral electrophoresis buffer for 30 min. Horizontal electrophoresis was performed at 4°C in low light conditions for 45 min at 21 V. Following the electrophoresis protocol, the slides were next immersed in DNA Precipitation Solution for 30 min and 70% ethanol for 30 min at room temperature. Each slide was dried with air and stained with 1x SYBR^®^ Gold Staining Solution (Trevigen, Gaithersburg, MD, USA) in the dark. All slides were washed with water and air dried. Samples were visualized using an EVOS FL Auto microscope (Life Technology, Carlsbad, CA, USA). DNA damage was quantified by evaluating both tail length (defined as the length of DNA migration and is related to DNA fragment size, calculated from the center of the cell and reported in micrometers) and tail moment (determined by tail length times the fraction of DNA in the tail). At least 50 random cells were scored per sample. Images were analyzed by Open Comet plugin in ImageJ 1.52e, (accessed 1 August 2018) for various comet parameters [14].

### 2.4. Clonogenic Assay

LLC cells were treated with 54.5 µg of either SGNP or BGNP for 48 h followed by 2 Gy radiation alone and combined with NP treatment using a Rad Source RS-2000 Biological Research Irradiator (Rad Source Technologies, Buford, GA, USA). Thirty minutes after irradiation cells were trypsinized and approximately 200–500 cells from each sample were plated in triplicate in 6-well plates. After 10–15 days, the colonies were stained with 0.5% crystal violet solution in 50% methanol. Clonogenic efficiency was measured by % area and/or % intensity through colony area plugin (ImageJ) [15].

### 2.5. In-Vitro Cellular Uptake of GNPs

To assess internalization of the GNPs by the LLC cells, cells were first seeded in 6-well plates at a concentration of 3 × 10^5^ cells/well with 4 mL of complete media. 50 µL of each gold nanoparticle sample were added from a solution of ~4 mg/mL to each well. Triplicate wells were treated for each sample type and allowed to incubate for 24 h at 37 °C and 5% humidity in a HERAcell 150i CO_2_ incubator (Thermo Fisher Scientific, Waltham, MA, USA). After incubation, the wells were washed three times with 1x Phosphate Buffered Saline (PBS) (Thermo Fisher Scientific, Waltham, MA, USA), and the cells detached using 0.25% trypsin–0.53 mM EDTA solution (Thermo Fisher Scientific, Waltham, MA USA) at a volume of 0.5 mL per well. Next, 1 mL of complete media was added, and the cells were counted and centrifuged at 100 G for 5 min, the supernatant removed, and the samples fixed with 1 mL of 4% paraformaldehyde (Electron Microscopy Sciences, Hatfield, PA, USA). Samples were then washed in 0.1 M PBS for 10 min three times, post-fixation treated with 2% osmium tetroxide (OsO_4_) in cacodylate buffer for 2 h at room temperature, washed again in 0.1 M PBS for 10 min three times, and dehydrated with a graded series of ethanol (30%, 50%, 70%, 90%) 10 min each, followed by 90% acetone for 10 min, and 100% acetone for 15 min three times. Pre-inclusion in resin / 100% acetone (1:1) for 2 h, pre-inclusion in resin / 100% acetone (2:1) overnight, and pre-inclusion in 100% resin for 3 h was next followed by embedding in 100% resin using flat embedding molds. Samples were placed in a 60 °C oven for 48 h for polymerization. 100 nm ultrathin sections were generated using a diamond knife and the sections mounted on copper grids (200 mesh) (Ted Pella, Inc., Redding, CA, USA). The ultrathin sections were stained with uranyl acetate and lead citrate and imaged using a FEI Nova NanoSEM 230 (FEI Co., Hillsboro, Oregon, USA) at STEM mode at 15 KV vacuum in the bright field setting.

### 2.6. Statistical Analysis

GraphPad Prism 8 software, (accessed on 30 October 2018) was used for all statistical analyses. Data are expressed as the median with interquartile range for comet assay and mean ± SD for the clonogenic assay. Asterisks denote *p*-values in the figures and sample sizes are included in each figure legend. One-way ANOVA was used to determine statistical significance.

## 3. Results and Discussion

### 3.1. Characterization and Physicochemical Properties of the SGNPs and BGNPs

**Figure 1:**
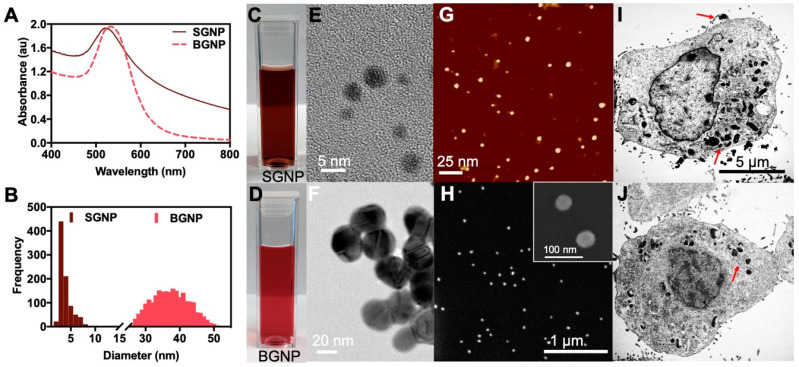
(**A**) Absorbance spectra of the small (SGNP) and big (BGNP) gold nanoparticles with an absorbance maxima occurring around 519 nm and 534 nm, respectively. (**B**) Size distributions of SGNP (average particle size of 3.86 ± 1.27 nm) and BGNP (average particle size 37.39 ± 5.52 nm) for over 300 particles. Optical micrograph of (**C**) SGNP and (**D**) BGNP. TEM images of (**E**) SGNP and (**F**) BGNP. (**G**) AFM image of the SGNP and (**H**) SEM image of the BGNP. (**I**) SGNP and (**J**) BGNP internalized in LLC cells. Red arrows highlight particle clusters within the vacuoles of the cell.

Two different-sized gold nanoparticles small (SGNP) and big (BGNP) were synthesized (Figure 1) and investigated for radio-sensitization effects in vitro. While particles of both sizes displayed similar optical absorption spectra (Figure 1A), they appeared different in color to the visible eye (Figure 1C,D). The larger particle size resulted in a shift in the absorbance maximum to a higher wavelength at 534 nm, while the peak from the smaller particles was observed at 519 nm. The particles measured a ten-fold difference in diameter (Figure 1B,E–H). At this size range, the NPs can be internalized by LLC cells via endocytosis into cells (Figure 1I,J). The particle clusters remain within vacuoles within the cell (highlighted by red arrows). Even without a targeting moiety, macropinocytosis of the SGNP can be clearly seen in the top right of Figure 1I. This is one of four types of endocytosis pathways and is a non-specific process to internalized fluids and particles together into the cells [16]. This observed efficiency in penetrating cells is one of the unique properties of GNPs [17], which we choose to exploit here for radiotherapy.

### 3.2. Effects of Gold Nanoparticles (SGNP and BGNP) with Radiation on DNA Damage in LLC Cells

**Figure 2:**
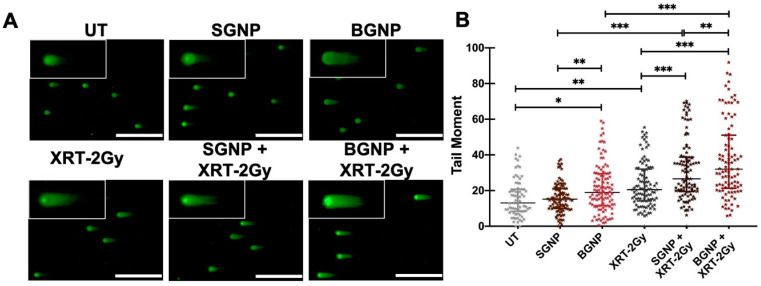
Neutral comet assay for LLC cells. Cells were untreated (UT), treated with small gold nanoparticles (SGNP) or big gold nanoparticles (BGNP), 2 Gy X-ray irradiation (XRT-2 Gy), or a combination of 2 Gy and either small or big gold nanoparticles. (**A**) Scale bar represents 400 µm. (**B**) The horizontal line shows the median and the vertical line shows the interquartile range. At least 50 random cells were scored per sample. A one-way ANOVA was performed to determine statistical significance (*p < 0.033, **p < 0.002, and ***p < 0.001).

The comet assay offers a robust technique to evaluate DNA damage in cells and has been broadly used to measure both DNA damage and repair in vitro after genotoxic stress [18]. Once an electric field is applied, denatured and cleaved DNA fragments migrate out of the nucleoid with more-damaged DNA migrating faster, yielding a “comet” tail shape. For a neutral comet assay, damage is assessed through double-stranded breaks in DNA. Figure 2A shows the visualization of a neutral comet assay by epifluorescence microscopy performed on untreated (UT) LLC cells and cells treated with SGNPs and BGNPs only, irradiated (XRT-2 Gy) cells, and combined treatment of radiation with SGNPs or BGNPs. It should be noted that the amount of gold incubated with the cells for both the SGNP and BGNP treatment groups was kept constant (54.5 µg/well in a 6-well plate with a surface area of 9 cm^2^ per well). Undamaged DNA remains in the head of the “comet”, and the tail represents the amount of damaged DNA (or charged DNA) that migrates in an electric field. A dose of 2 Gy was chosen as it is not only a typical dose used in the literature [19,20,21] but also showed significance in modifying the tail moment when compared to the untreated cells with evidence of a synergistic effect when GNPs were present.

When plotted as a function of tail moment, no significant difference was observed between the UT and SGNP groups, however, a significant (*p < 0.033) increase in DNA damage was found in the BGNP group when compared to UT cells. Researchers have noticed-size dependent toxicity of gold nanoparticles, [22] but it is dependent on assay type, cell line, and nanoparticle properties, leading to conflicting results. In fact, we saw evidence of this as, when compared to untreated cells, treatment with BGNPs alone (~40 nm) showed a cytotoxic effect by increasing the median tail moment value from 12.5 to 19.5.

In another study, the comparison of different particle sizes (30, 50, and 90 nm) and concentrations (1, 5, 10 μg/mL) of commercially available gold nanoparticles were assessed using a comet assay in hepatoma and leukemia cell lines. The authors found significant difference in DNA damage with the gold nanoparticles compared to vehicle control, however, the size and concentration of the gold nanoparticles did not increase the DNA damage significantly. Furthermore, in this paper, the authors did not evaluate the combined effects of radiation with gold nanoparticles [23]. As evident in our work, a significant difference in DNA damage was observed (**p < 0.002) between the UT and radiation only (XRT-2 Gy) treated group. Further, synergistic increase of DNA damage (***p < 0.001) was observed in combinatorial treatment of radiation with either of SGNPs and BGNPs (SGNP XRT-2 Gy or BGNP XRT-2 Gy). When both particles are compared to each other, a significant difference was seen in both irradiated and non-irradiated groups. We found that both particles sizes significantly radio-sensitize the LLC cells, and that the bigger nanoparticles induced greater DNA damage compared to the smaller particles (as evidenced by the higher median value of comet tail moment) in both the irradiated and non-irradiated groups. It is not surprising that exposure to radiation resulted in evidence of a higher tail moment compared to the untreated group, since ionizing radiation is known to produce double-stranded breaks due to the physico-chemical interaction with cellular DNA [18]. However, overall, the greater tail moment for the cells irradiated in the presence of gold nanoparticles indicates that for these two treatment groups, DNA damage was more significant with respect to radiation alone.

### 3.3. Effects of Gold Nanoparticles (SGNP and BGNP) with Radiation on LLC Cell Survival

We experimentally evaluated the effects of two different sizes of gold nanoparticles (~4 nm and ~40 nm) on cell survival as we found size-dependent comparisons for radio-sensitization lacking in the current literature. For example, a study by Yang et al. [24] evaluated the effects of radiation with 10 nm gold nanoparticles on cell survival in the breast cancer cell line MDA-MB-231, where the authors reported a 19 ± 6% (*p* < 0.05) reduction in survival when compared to radiation only. However, although they discuss treatment with 2-nm-sized particles, effects were only theoretically assessed through Monte Carlo simulation. Further, the modeling resulted in no effect on cell survival with the smaller 2 nm gold nanoparticles, indicating a need for experimental evaluation.

**Figure 3:**
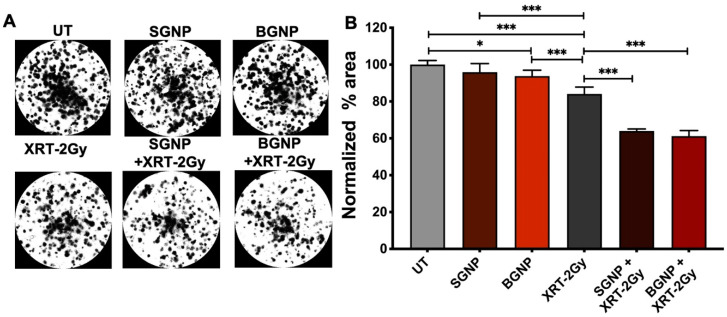
(**A**) Clonogenic assay performed in vitro with LLC to measure the survival potential after the various treatment paradigms. (**B**) Normalized percentage area for the different treatment groups. Cells from each sample were plated in triplicate. A one-way ANOVA was performed to determine statistical significance (*p < 0.033, **p < 0.002, and ***p < 0.001).

In our studies, clonogenic assessment (Figure 3) showed that the ability of cells to replicate decreased significantly after treatment with BGNPs and radiation. In contrast, combinatorial treatment of radiation with both particles further significantly reduced the cell survival. However, we did not observe any significant difference in cell survival between SGNPs and BGNPs due to difference in particle size. We hypothesize that this maybe because any significant differences in damage created due to particle size may be repaired during the standard 14-day incubation for cell survival assessment. Changing the incubation time and increasing the radiation dose may better reflect size-dependent effects of DNA damage to cell survival. While the presence of GNPs did not visibly alter the proliferation of the cells into colonies, a reduction in the number of cells can be seen when treated with irradiation alone and combination of GNPs and irradiation (Figure 3A).

When the data is represented as a function of the normalized percentage area (Figure 3B), statistical significance can be evaluated for the different groups. The three control groups, UT, SGNP, and BGNPs, all show a normalized percent area within error of each other. In the presence of irradiation, however, significance (***p < 0.001) is observed between the UT and XRT-2 Gy groups as well as significance (***p < 0.001) between the SGNP and XRT-2 Gy and BGNP and XRT-2 Gy groups is observed. In combinatorial treatment of radiation with either of SGNPs and BGNPs (SGNP XRT-2 Gy or BGNP XRT-2 Gy) a significance of (***p < 0.001) is found when compared with the XRT-2 Gy radiation alone, again demonstrating a synergistic effect when combined. When combined with irradiation, the presence of the particles decreased the percentage area by 36% and 39% for the SGNP and BGNP, respectively, as compared to the radiation alone (XRT-2 Gy) group.

## 4. Conclusions

In summary, GNPs of two different dimensions were fabricated to examine if GNPs could act as effective radio-sensitizers in vitro in a non-small cell lung cancer model. Comet and clonogenic assays performed with Lewis lung carcinoma cells demonstrated that both sizes of the GNPs showed significant radio-sensitization and reduced cell survival after treatment. Greater significance was observed in vitro for inducing DNA damage with the BGNP treatment group supported by the higher median value of comet tail moment. However, we did not find any significant difference in cell survival due to particle size in the irradiated group, which could be enhanced by using a higher dose of radiation as 2 Gy may be considered a lower dose for this cell line. These GNPs can be functionalized with various moieties, such as bovine serum albumin, and injected into solid tumors in mice where their distribution and retention can be assessed by CT [25]. Surface functionalization of GNPs can also be used to help with gene silencing in cancer cells [26] or specific tumor cell targeting [27]. Local administration of agents intratumorally combined with radiation is proving beneficial in advanced stages of cancer treatment [28,29,30]. Further in-vivo evaluation of the effects of GNPs on radiation enhancement may help with the translation of these particles toward use in a clinical setting.

## References

[1] Arvizo R., Bhattacharya R., Mukherjee P. (2010). Gold nanoparticles: Opportunities and challenges in nanomedicine. Expert Opin. Drug Deliv..

[2] Huang X., El-Sayed I.H., Qian W., El-Sayed M.A. (2006). Cancer Cell Imaging and Photothermal Therapy in the Near-Infrared Region by Using Gold Nanorods. J. Am. Chem. Soc..

[3] Popovtzer R., Agrawal A., Kotov N.A., Popovtzer A., Balter J., Carey T.E., Kopelman R. (2008). Targeted gold nanoparticles enable molecular CT imaging of cancer. Nano Lett..

[4] Her S., Jaffray D.A., Allen C. (2017). Gold nanoparticles for applications in cancer radiotherapy: Mechanisms and recent advancements. Adv. Drug Deliv. Rev..

[5] Babaei M., Ganjalikhani M. (2014). The potential effectiveness of nanoparticles as radio sensitizers for radiotherapy. Bioimpacts.

[6] Leung M.K.K., Chow J.C.L., Chithrani B.D., Lee M.J.G., Oms B., Jaffray D.A. (2011). Irradiation of gold nanoparticles by x-rays: Monte Carlo simulation of dose enhancements and the spatial properties of the secondary electrons production: Monte Carlo simulation on gold nanoparticles. Med. Phys..

[7] Brun E., Sanche L., Sicard-Roselli C. (2009). Parameters governing gold nanoparticle X-ray radiosensitization of DNA in solution. Colloids Surf. B Biointerfaces.

[8] Butterworth K.T., Wyer J.A., Brennan-Fournet M., Latimer C.J., Shah M.B., Currell F.J., Hirst D.G. (2008). Variation of Strand Break Yield for Plasmid DNA Irradiated with High- *Z* Metal Nanoparticles. Radiat. Res..

[9] Duff D.G., Baiker A., Edwards P.P. (1993). A new hydrosol of gold clusters. 1. Formation and particle size variation. Langmuir.

[10] Wang H., Levin C.S., Halas N.J. (2005). Nanosphere Arrays with Controlled Sub-10-nm Gaps as Surface-Enhanced Raman Spectroscopy Substrates. J. Am. Chem. Soc..

[11] Levin C.S., Janesko B.G., Bardhan R., Scuseria G.E., Hartgerink J.D., Halas N.J. (2006). Chain-Length-Dependent Vibrational Resonances in Alkanethiol Self-Assembled Monolayers Observed on Plasmonic Nanoparticle Substrates. Nano Lett..

[12] Levin C.S., Kundu J., Janesko B.G., Scuseria G.E., Raphael R.M., Halas N.J. (2008). Interactions of Ibuprofen with Hybrid Lipid Bilayers Probed by Complementary Surface-Enhanced Vibrational Spectroscopies. J. Phys. Chem. B.

[13] Campeau E., Ruhl V.E., Rodier F., Smith C.L., Rahmberg B.L., Fuss J.O., Campisi J., Yaswen P., Cooper P.K., Kaufman P.D. (2009). A Versatile Viral System for Expression and Depletion of Proteins in Mammalian Cells. PLoS ONE.

[14] Gyori B.M., Venkatachalam G., Thiagarajan P.S., Hsu D., Clement M.-V. (2014). OpenComet: An automated tool for comet assay image analysis. Redox Biol..

[15] Guzmán C., Bagga M., Kaur A., Westermarck J., Abankwa D. (2014). ColonyArea: An ImageJ Plugin to Automatically Quantify Colony Formation in Clonogenic Assays. PLoS ONE.

[16] Park J.H., Oh N. (2014). Endocytosis and exocytosis of nanoparticles in mammalian cells. Int. J. Nanomed..

[17] Xie X., Liao J., Shao X., Li Q., Lin Y. (2017). The Effect of shape on Cellular Uptake of Gold Nanoparticles in the forms of Stars, Rods, and Triangles. Sci. Rep..

[18] Garaj-Vrhovac V., Kopjar N. (2003). The alkaline Comet assay as biomarker in assessment of DNA damage in medical personnel occupationally exposed to ionizing radiation. Mutagenesis.

[19] Dunne A.L., Price M.E., Mothersill C., McKeown S.R., Robson T., Hirst D.G. (2003). Relationship between clonogenic radiosensitivity, radiation-induced apoptosis and DNA damage/repair in human colon cancer cells. Br. J. Cancer.

[20] Palyvoda O., Polanska J., Wygoda A., Rzeszowska-Wolny J. (2003). DNA damage and repair in lymphocytes of normal individuals and cancer patients: Studies by the comet assay and micronucleus tests. Acta Biochim. Pol..

[21] Kurashige T., Shimamura M., Nagayama Y. (2016). Differences in quantification of DNA double-strand breaks assessed by 53BP1/γH2AX focus formation assays and the comet assay in mammalian cells treated with irradiation and N-acetyl-L-cysteine. J. Radiat. Res..

[22] Alkilany A.M., Murphy C.J. (2010). Toxicity and cellular uptake of gold nanoparticles: What we have learned so far?. J. Nanoparticle Res..

[23] Ávalos A., Haza A.I., Mateo D., Morales P. (2018). In vitro and in vivo genotoxicity assessment of gold nanoparticles of different sizes by comet and SMART assays. Food Chem. Toxicol..

[24] Yang C., Bromma K., Sung W., Schuemann J., Chithrani D. (2018). Determining the Radiation Enhancement Effects of Gold Nanoparticles in Cells in a Combined Treatment with Cisplatin and Radiation at Therapeutic Megavoltage Energies. Cancers.

[25] Terracciano R., Sprouse M.L., Wang D., Ricchetti S., Hirsch M., Ferrante N., Butler E.B., Demarchi D., Grattoni A., Filgueira C.S. Intratumoral Gold Nanoparticle-Enhanced CT Imaging: An in Vivo Investigation of Biodistribution and Retention. Proceedings of the 2020 IEEE 20th International Conference on Nanotechnology (Ieee-Nano).

[26] Shen J., Kim H.C., Mu C., Gentile E., Mai J., Wolfram J., Ji L., Ferrari M., Mao Z., Shen H. (2014). Multifunctional Gold Nanorods for siRNA Gene Silencing and Photothermal Therapy. Adv. Healthc. Mater..

[27] Jaganathan H., Mitra S., Srinivasan S., Dave B., Godin B. (2014). Design and In Vitro Evaluation of Layer by Layer siRNA Nanovectors Targeting Breast Tumor Initiating Cells. PLoS ONE.

[28] Liu H.C., Viswanath D.I., Pesaresi F., Xu Y., Zhang L., Di Trani N., Paez-Mayorga J., Hernandez N., Wang Y., Erm D.R. (2020). Potentiating anti-tumor efficacy through radiation and sustained intratumoral delivery of anti-CD40 and anti-PDL1. Int. J. Radiat. Oncol. Biol. Phys..

[29] Chua C.Y.X., Ho J., Susnjar A., Lolli G., Di Trani N., Pesaresi F., Zhang M., Nance E., Grattoni A. (2020). Intratumoral Nanofluidic System for Enhancing Tumor Biodistribution of Agonist CD40 Antibody. Adv. Ther..

[30] Chua C.Y.X., Ho J., Demaria S., Ferrari M., Grattoni A. (2020). Emerging Technologies for Local Cancer Treatment. Adv. Ther..

